# Revising pathogenesis of *AP1S1*-related MEDNIK syndrome: a missense variant in the *AP1S1* gene as a causal genetic lesion

**DOI:** 10.1007/s00109-024-02482-0

**Published:** 2024-09-13

**Authors:** Marketa Rackova, Rafael Mattera, Michael Svaton, Filip Fencl, Veronika Kanderova, Karolina Spicakova, Sang Yoon Park, Ondrej Fabian, Miroslav Koblizek, Eva Fronkova, Juan S. Bonifacino, Karolina Skvarova Kramarzova

**Affiliations:** 1 CLIP, Department of Pediatric Hematology and Oncology, Second Faculty of Medicine, Charles University and University Hospital Motol, Prague, Czech Republic; 2 Section on Intracellular Protein Trafficking, Neurosciences and Cellular and Structural Biology Division, Eunice Kennedy Shriver National Institute of Child Health and Human Development, National Institutes of Health, Bethesda, MD USA; 3 Department of Pediatrics, Second Faculty of Medicine, Charles University and University Hospital Motol, Prague, Czech Republic; 4 Department of Pathology and Molecular Medicine, Second Faculty of Medicine, Charles University and University Hospital Motol, Prague, Czech Republic

**Keywords:** Coatopathies, MEDNIK, *AP1S1*, Congenital diarrhea, Missense variants

## Abstract

**Abstract:**

MEDNIK syndrome is a rare autosomal recessive disease characterized by mental retardation, enteropathy, deafness, peripheral neuropathy, ichthyosis, and keratoderma, and caused by variants in the adaptor-related protein complex 1 subunit sigma 1 (*AP1S1*) gene. This gene encodes the σ1A protein, which is a subunit of the adaptor protein complex 1 (AP-1), a key component of the intracellular protein trafficking machinery. Previous work identified three *AP1S1* nonsense, frameshift and splice-site variants in MEDNIK patients predicted to encode truncated σ1A proteins, with consequent AP-1 dysfunction. However, two *AP1S1* missense variants (c.269 T > C and c.346G > A) were recently reported in patients who presented with severe enteropathy but no additional symptoms of MEDNIK. This condition was described as a novel non-syndromic form of congenital diarrhea caused specifically by the *AP1S1* missense variants. In this study, we report two patients with the same c.269 T > C variant, who, contrary to the previous cases, presented as complete MEDNIK syndrome. These data substantially revise the presentation of disorders associated with *AP1S1* gene variants and indicate that all the identified pathogenic *AP1S1* variants result in MEDNIK syndrome. We also provide a series of functional analyses that elucidate the impact of the c.269 T > C variant on σ1A function, contributing to a better understanding of the molecular pathogenesis of MEDNIK syndrome.

**Key messages:**

- A missense *AP1S1* c.269 T > C (σ1A L90P) variant causes full MEDNIK syndrome.
- The σ1A L90P variant is largely unable to assemble into the AP-1 complex.
- The σ1A L90P variant fails to bind [DE]XXXL[LI] sorting motifs.
- The σ1A L90P variant results in loss-of-function of the protein.

**Supplementary Information:**

The online version contains supplementary material available at 10.1007/s00109-024-02482-0.

## Introduction

**Fig.1:**
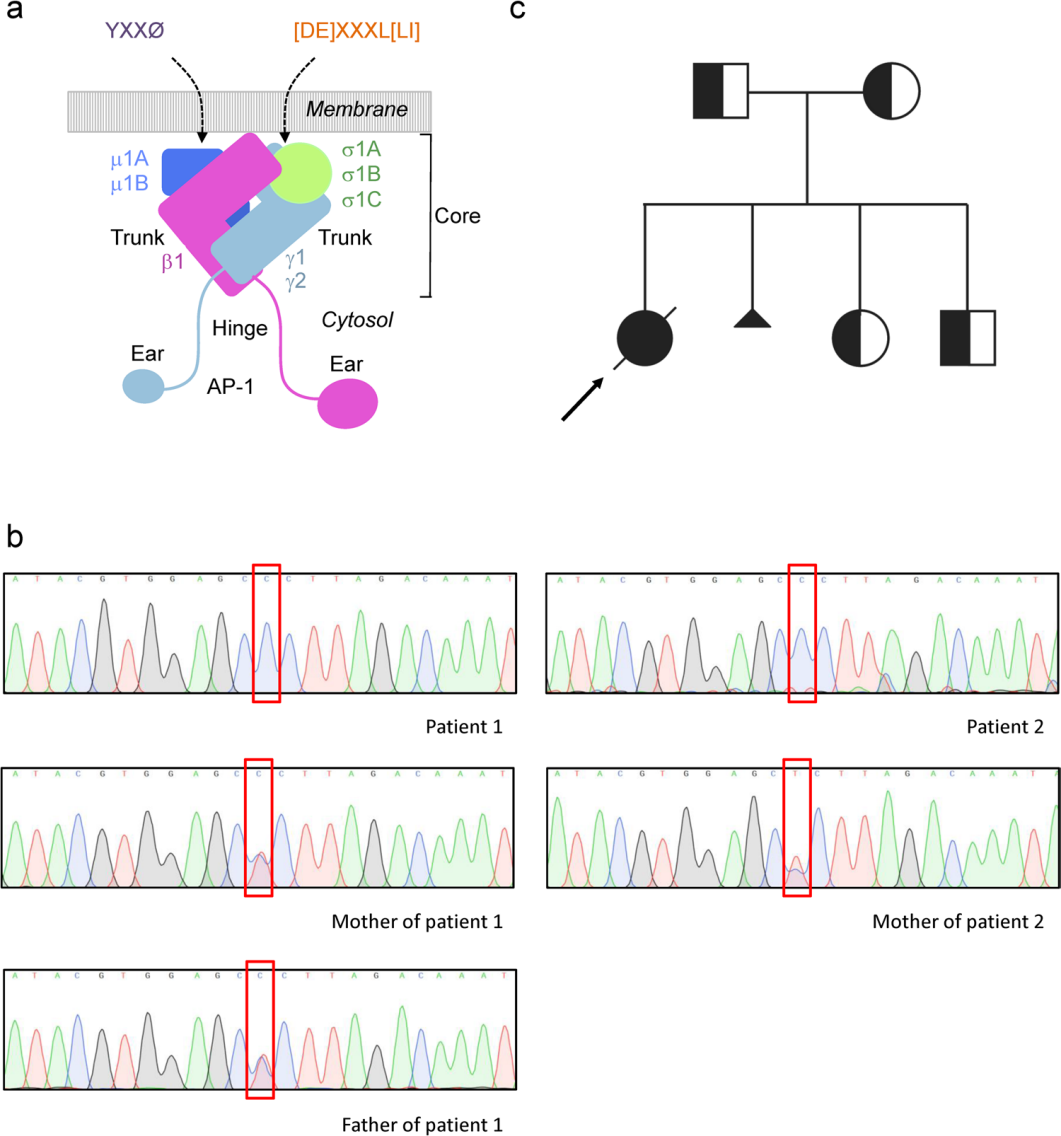
Structure of the AP-1 complex and genetic analysis. **a** Schematic representation of the AP-1 complex depicting its γ, β1, μ1 and σ1 subunits. The γ, μ1 and σ1 subunits occur as multiple isoforms encoded by different genes, namely, γ1 and γ2, μ1A and μ1B, and σ1A, σ1B and σ1C, respectively. Also shown are the trunk, hinge and ear domains of γ and β1. The trunk domains of γ and β1 together with the μ1 and σ1 subunits constitute the core of the AP-1 complex. The indicated tyrosine-based YXXØ and dileucine-based [DE]XXXL[LI] sorting signals in the cytosolic tails of transmembrane protein cargos are recognized by the AP-1 μ1 subunit and the γ-σ1 hemicomplex, respectively. **b** Detection of *AP1S1* variant c.269 T > C. Representative sequence traces from patient 1 and her mother and father, and from patient 2 and his mother. **c** Pedigree of patient 1. Arrow indicates proband, square—male, circle—female, diagonal line—deceased, small triangle—termination of pregnancy

Intracellular trafficking is a complex dynamic process that maintains cellular homeostasis by tightly regulating the exchange of molecules between distinct cellular compartments. A large group of monogenic disorders arising from defects in intracellular trafficking has been described [1]. These include coatopathies, a category of diseases associated with variants in genes encoding membrane coat proteins, including subunits of heterotetrameric adaptor protein (AP) complexes [2, 3]. Five AP complexes named AP-1 through AP-5 have been identified, each having a specific sorting function at a distinct intracellular location [2, 3]. AP-1 is a clathrin-associated complex that mediates sorting of transmembrane proteins between the *trans*-Golgi network (TGN) and endosomes in all cells, as well as polarized sorting to the basolateral surface of epithelial cells and the somatodendritic domain of neurons [2, 3]. AP-1 consists of two large γ and β1 subunits, a medium-sized μ1 subunit, and a small σ1 subunit [2, 3] (Fig. 1a). AP-1-mediated sorting involves recognition of signals in the cytosolic tails of transmembrane proteins, including tyrosine-based YXXØ motifs binding to the μ1 subunit and dileucine-based [DE]XXXL[LI] motifs binding to the γ-σ1 hemicomplex (X means any amino acid and Ø a bulky hydrophobic amino acid) [4–7] (Fig. 1a). In mammals, the γ, μ1 and σ1 subunits occur as multiple isoforms encoded by different genes (i.e., paralogs) (Fig. 1a). The human σ1 subunit, in particular, exists as σ1A, σ1B and σ1C isoforms encoded by the *AP1S1*, *AP1S2* and *AP1S3* genes, respectively (Fig. 1a). The physiological relevance for the existence of AP subunit isoforms is underscored by the diverse phenotypes of model organisms with variants in the different isoforms as well as the identification of distinct human disorders caused by variants in these genes [2, 3].

The first pathogenic variant in the *AP1S1* gene (c.301-2A > G, splice-site variant) was described in 2008 in four French-Canadian families from Quebec, causing a rare autosomal recessive disease characterized by mental retardation, enteropathy, deafness, neuropathy, ichthyosis, and keratoderma, and named MEDNIK syndrome as an acronym of these clinical features [8, 9]. The typical manifestations of MEDNIK syndrome reflect the described function of the AP-1 complex, as they involve tissues with a core component of polarized cells, such as neurons and epithelial cells. Since then, an *AP1S1* c.356_365insG frameshift variant was described in two unrelated patients from Sephardic Jewish and Turkish families who presented with identical symptoms [10, 11]. Furthermore, another *AP1S1* variant (c.186 T > G, nonsense) was recently identified in a MEDNIK patient from a Mexican family [12]. All these variants were proven or predicted to result in decreased or missing expression of the variant protein, thus leading to σ1A loss of function (LOF).

In 2020, Klee et al. described two novel variants in the *AP1S1* gene (missense variants c.269 T > C and c.346G > A) in three patients with congenital diarrhea, who presented distinctly from the previously reported MEDNIK cases [13]. The most prominent symptom of the affected individuals was enteropathy, whereas deafness, neuropathy, and skin disorders were not observed. The authors suggested that, contrary to the previously reported variants in the *AP1S1* gene, these missense *AP1S1* variants caused a non-syndromic form of congenital diarrhea [13]. In this report, we present two additional patients with the *AP1S1* c.269 T > C variant from two unrelated families who, similarly to the cases described by Klee et al., presented with severe neonatal onset enteropathy. However, both patients also exhibited other symptoms typical of MEDNIK syndrome, which challenges the hypothesis that *AP1S1* c.269 T > C causes a unique disease differing significantly from MEDNIK syndrome. We also performed functional analyses to further elucidate the impact of the c.269 T > C variant on σ1A function. These analyses showed that this σ1A variant was expressed at about half the levels of wild-type (WT) σ1A and was virtually incapable of assembling into the AP-1 complex. As a consequence, the amount of AP-1 at the TGN/endosomes was greatly reduced, and the recognition of [DE]XXXL[LI] signals was completely abrogated. Altogether, we revise the characterization of the disorders associated with *AP1S1* gene variants and provide novel insights into the molecular pathogenesis of MEDNIK syndrome.

## Materials and methods

### Patients

In accordance with the declaration of Helsinki and the Institutional Review Board of the Second Faculty of Medicine, Charles University and University Hospital Motol, participants in this study provided written consent for the genetic study, research investigations and publication.

### Cell lines

HeLa and HEK293T cells were grown in Dulbecco’s Modified Eagle’s Medium (DMEM, Gibco, Billings, MT, USA) supplemented with 10% fetal bovine serum (FBS) (Corning, NY, USA), 2 mM L-glutamine (Gibco), 100 units/ml of penicillin and 100 μg/ml streptomycin (Gibco) at 37 °C in a 5% CO_2_ atmosphere. WT, σ1B-KO (AP1S2_15020-04) and σ1C-KO (AP1S3_12627-06) HAP1 cells were purchased from Horizon Discovery (Cambridge, UK). HAP1 cells were grown in Iscove’s Modified Dulbecco’s Medium (IMDM, Gibco) supplemented with 10% FBS, 100 units/ml of penicillin and 100 μg/ml streptomycin at 37 °C in a 5% CO_2_ atmosphere. Cells were transiently transfected as previously described [14], and used for immunoblotting, co-immunoprecipitation, and immunofluorescence experiments as described below.

### Nucleic acid isolation

DNA from patient/healthy control samples was isolated using QIAamp DNA Blood Mini Kit (QIAGEN, Hilden, Germany). Total RNA was isolated from formalin-fixed paraffin-embedded (FFPE) samples of intestinal tissue using High Pure FFPET RNA Isolation Kit (Roche, Basel, Switzerland). Samples of duodenum and rectum from six patients with Hirschsprung disease (unaffected part), intermittent diarrhea without endoscopic or histopathological findings, or individuals screened for colon cancer were used as controls.

### Whole exome sequencing

Whole exome sequencing was performed on a NextSeq 500 instrument (Illumina, San Diego, CA, USA) using the SureSelectXT Human All Exon V6 + UTRs kit (Agilent Technologies, Santa Clara, CA, USA) for library preparation. Sequencing reads were aligned to the hg19 human reference genome with BWA [15] and variant calling was performed using VarScan2 [16] and SAMtools [17]. Variants were annotated and filtered using the Ingenuity Variant Analysis (QIAGEN). A novel homozygous variant (c.269 T > C) in the *AP1S1* gene (NM_001283.5:c.269 T > C) was identified based on the deleteriousness prediction using CADD [18]. Homozygosity mapping analysis from the sequencing data was performed using the AutoMap tool [19]. The presence of the c.269 T > C variant in the patients was verified by Sanger sequencing (Eurofins Genomics, Ebersberg, Germany) and the variant segregation in the families determined from DNA available for analysis.

### Additional methods

Detailed descriptions of immunoprecipitation and immunoblotting, immunofluorescence microscopy, immunohistochemistry, generation of recombinant DNA constructs and yeast three-hybrid assays, gene expression assays and generation of knock-out of AP-1 σ1-subunit genes by CRISPR/Cas9, are available in the Supplementary File.

## Results

### Clinical characteristics of the patients

**Patient 1** is a female born from the first pregnancy to healthy parents of Romani origin at 34 weeks of gestation, with birth weight and length in the 50th percentile. In her first week of life, she presented with frequent watery stools leading to severe dehydration accompanied by mild metabolic acidosis and hypoglycemia requiring parenteral nutrition. Apart from electrolyte disturbances caused by dehydration, her laboratory findings showed progressive conjugated hyperbilirubinemia, which later resolved spontaneously. Liver enzymes were only transiently elevated. Despite all the therapeutic efforts, her clinical status progressively deteriorated, and she died of septic shock at 18 weeks of age (Table 1).

**Patient 2** is a male born from the 12th pregnancy and 7th parity to another couple of healthy parents of Romani origin at 37 weeks of gestation, with birth weight and length in the 22nd percentile. Four days after birth, he presented with frequent watery acholic stools and malabsorption, leading to severe dehydration requiring parenteral nutrition. Histopathological findings in the small bowel and colon were similar, revealing mild to moderate chronic focally active enteritis and colitis with the presence of numerous apoptotic bodies in the crypt epithelium and disrupted mucosal architecture, including distorted crypts and shortened villi. Unlike patient 1, the symptoms of his enteropathy gradually subsided over time, and by the end of the second year of life, he was switched to full enteral nutrition. This patient also showed signs of renal disease characterized by progressive proteinuria with dominating tubular component (up to 205 mg/mmol creatinine), and mild hepatopathy (intermittent elevation of liver enzymes with preserved function of protein synthesis and elimination) (Table 1). He died at the age of 5.5 years due to aspiration in home care.

**Table 1:** Clinical characteristics of patients with the *AP1S1* c.269 T > C variant

	Patient 1	Patient 2	Klee et al. pt.1	Klee et al. pt.2
Gender	F	M	F	F
Age at death	18 weeks	5.5 years	4 weeks	3 weeks
Enteropathy	+	+	+	+
Deafness	NA	+	-	-
Ichthyosis	+	+	-	-
Keratoderma	+	+	-	-
Hepatopathy	+	transient	NA	NA
Serum copper*	4.8 μmol/l (↓)	1.7–7.5 μmol/l (↓)	NA	NA
Serum ceruloplasmin^#^	0.06–0.17 g/l (↓)	0.06–0.16 g/l (↓)	NA	NA
VLCFA^φ^	NA	within range	NA	NA
Bilirubin max	total: 228 μmol/l, conjugated: 135 μmol/l	total: 223 μmol/l, conjugated: 39 μmol/l	NA	NA

^*^Reference range 10.3—21.4 μmol/l, ^**#**^Reference range 0.18—0.45 g/l, ^φ^Very-long-chain fatty acids

↓ Below the reference range (lower reference limit)

NA – not available

Both patients had ichthyosis and keratoderma notable already in the neonatal period. Otoacoustic emissions repeatedly failed to be detected in both patients and patient 2 later showed clinical signs of deafness. Neurological symptoms, characteristic of MEDNIK syndrome, were not initially observed. However, patient 2 subsequently exhibited global developmental delay, intellectual disability, and peripheral neuropathy. Characteristic disturbances in copper metabolism were also present in the patients (Table 1). Both patients experienced several episodes of sepsis; the majority of them were linked to re-initiation of enteral nutrition. The spectrum of pathogens detected in blood cultures suggested either catheter-related infections or translocation of bacteria from the intestine. Furthermore, both patients suffered from recurrent venous thrombosis. The second patient also suffered from hypogammaglobulinemia requiring repeated immunoglobulin substitution.

### Genetic analysis

Whole exome sequencing (WES) was performed in both patients independently, and the results did not reveal any known monogenic enteropathy, inborn error of immunity, or metabolic disorder. Analysis of homozygous variants revealed a missense c.269 T > C (p.L90P) variant in the *AP1S1* gene (Fig. 1b) predicted to be highly deleterious (CADD: 28.5) and leading to a change of a highly conserved leucine residue (Supplementary Fig. 1a). This variant had not been reported at the time of our initial analysis but was later shown to be pathogenic by Klee et al. [13]. Parents and two siblings of patient 1 and the mother of patient 2 were found to be heterozygous carriers of the c.269 T > C variant by Sanger sequencing (Fig. 1b, c); the father of patient 2 did not give consent to genetic examination.

Of note, most of the previously described cases with *AP1S1* homozygous variants were from consanguineous couples. Although both families of our patients denied consanguinity, homozygosity mapping analysis of the WES data showed large runs of homozygosity in both patients 1 (140.13 Mb) and 2 (362.13 Mb), which is highly suggestive of parental blood relationships (Supplementary Fig. 1b). Furthermore, examination of the coding SNVs in the region surrounding the variant in both patients revealed an identical haplotype over a 300 kb-long region flanking the variant. The genotype of the patients in this region consists mostly of minor alleles with a population frequency of 0.08–4.8%, which suggests that the c.269 T > C variant is linked with this specific haplotype and has a founder nature.

### Molecular and functional characterization of AP-1 σ1A^L90P^

To assess the impact of the L90P variant on σ1A and the AP-1 complex we used a heterologous expression system involving transient expression of WT and variant σ1A cDNA constructs in human HeLa, HEK293T and HAP1 cells.

#### Decreased levels and assembly of σ1A^L90P^

**Fig.2:**
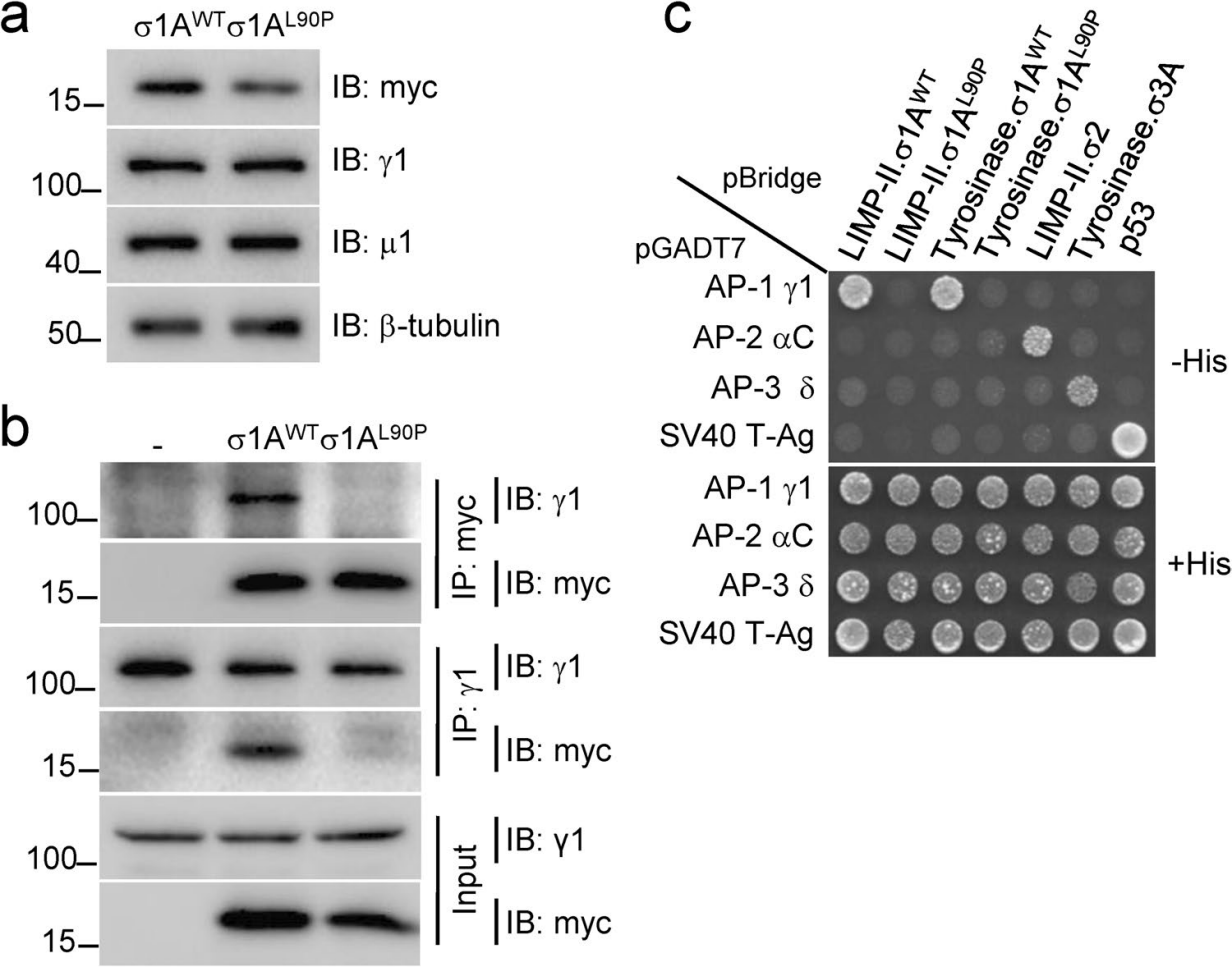
The AP-1 σ1A L90P substitution impairs assembly of the AP-1 complex and recognition of dileucine signals. **a** Decreased expression of myc-tagged σ1A L90P relative to WT myc-tagged σ1A expressed by transient transfection in HeLa cells and analyzed by SDS-PAGE and immunoblotting (IB). Blots of endogenous γ1, μ1 and β-tubulin are included as loading controls. **b** Impaired assembly of myc-tagged σ1A L90P into the AP-1 complex. HEK293T cells were transiently transfected with plasmids encoding either WT or L90P myc-tagged σ1A, and cell extracts were subjected to immunoprecipitation (IP) with anti-myc followed by SDS-PAGE and IB with anti-γ1 or anti-myc, or IP with anti-γ1 followed by SDS-PAGE and IB with anti-γ1 or anti-myc. Untransfected cells (-) were used as control. Notice that both permutations of IP and IB showed decreased co-immunoprecipitation of endogenous γ1 with myc-tagged σ1A L90P relative to myc-tagged WT σ1A (ranging from 0 to 13%, depending on the antibody combination; first and fourth blots from top). **c** Y3H assays showing lack of interaction of σ1A L90P with dileucine-based sorting signals. The AP-1 γ1, AP-2 αC and AP-3 δ subunits were subcloned in the Gal4 transcriptional activation domain (AD) vector pGADT7. The cytosolic tails of LIMP-II or tyrosinase and the indicated σ subunits were subcloned in the MCS1 and MCS2 of the Gal4 DNA binding domain (BD) vector pBridge, respectively. Transformants were plated on medium lacking leucine, tryptophan and methionine but containing histidine (+ His, bottom panel) to control for viability and loading, and on the same medium lacking histidine (-His, top panels) to detect protein interactions. The top panel shows the interaction of the γ1-σ1A hemicomplex with the dileucine motifs in the cytosolic tails of LIMP-II and tyrosinase (ERAPLI and ERQPLL, respectively [6]; and that the σ1A L90P substitution abrogates this interaction. Note the selective interaction of the LIMP-II and tyrosinase tails with the AP-1 γ1-σ1A hemicomplex but not with mismatched combinations of AP subunits. Additional controls in the assay include the interaction of the LIMP-II tail with AP-2 αC-σ2 and of the tyrosinase tail with AP-3 δ-σ3A (but not with mismatched combinations of AP subunits). Yeast co-transformation of pBridge-based constructs with a Gal4 AD-SV40 T-Ag fusion construct and of AD-AP subunit fusions with a Gal4 BD-p53 construct were used as negative controls. Co-transformants co-expressing AD-SV40 T-Ag and BD-p53 fusions provided a positive control for interactions

First, we examined the levels of the σ1A^WT^ and σ1A^L90P^ proteins appended with a C-terminal triple-myc tag in transfected HeLa cells [20]. We observed that σ1A^L90P^ was expressed at ~ 56% the levels of σ1A^WT^ (Fig. 2a), possibly due to destabilization and partial degradation of the protein. This effect is consistent with the expected disruption of the α-helix harboring leucine-90 by the conformationally restricted proline residue (Supplementary Fig. 1a). Next, we analyzed the impact of the L90P variant on the assembly of σ1A into the AP-1 complex. Since the σ1A subunit interacts most extensively with the γ1 subunit [21], we examined the effect of the L90P variant on the assembly of σ1A with γ1. To this end, we transfected HEK293T cells with plasmids encoding myc-tagged σ1A^WT^ or σ1A^L90P^, and examined the assembly of these constructs with endogenous γ1. Reciprocal co-immunoprecipitation experiments showed that assembly of γ1 with σ1A^L90P^ was reduced to 0–13% relative to σ1A^WT^ (Fig. 2b, first and fourth blots from top). The fact that the decrease in assembly was greater than the decrease in protein levels (Fig. 2a top blot and Fig. 2b bottom blot) indicated that the L90P substitution also impairs the interaction of σ1A with γ1, thus preventing proper assembly of the AP-1 complex.

#### Abrogation of [DE]XXXL[LI] motif recognition by σ1A^L90P^

Next, we used a yeast three-hybrid assay (Y3H) to examine the impact of the σ1A L90P variant on the recognition of dileucine-based, [DE]XXXL[LI] sorting motifs, which bind to a site including residues from both σ1A and γ1 [4, 6, 21]. The assays were performed by co-expression of different combinations of large and small subunits of AP-1, AP-2 and AP-3 with the [DE]XXXL[LI]-containing cytosolic tails of the lysosomal membrane protein LIMP-II and the melanosomal membrane protein tyrosinase [4, 6] (Fig. 2c). These experiments showed that σ1A^WT^, in combination with γ1 but not with the homologous AP-2 αC and AP-3 δ subunits, interacted with the cytosolic tail of LIMP-II and tyrosinase (Fig. 2c). Importantly, we observed that the L90P substitution completely abolished the interaction of the γ1-σ1A hemicomplex with the LIMP-II and tyrosinase tails in the Y3H assay (Fig. 2c). This result was consistent with the virtual inability of σ1A to assemble with γ1 (Fig. 2b). Because of the proximity of the L90P substitution to the [DE]XXXL[LI]-binding site (Supplementary Fig. 1a), this variant may also impair the ability of σ1A to participate in signal recognition.

#### Localization of the AP-1 complex in σ1A^L90P^-expressing cells

**Fig.3:**
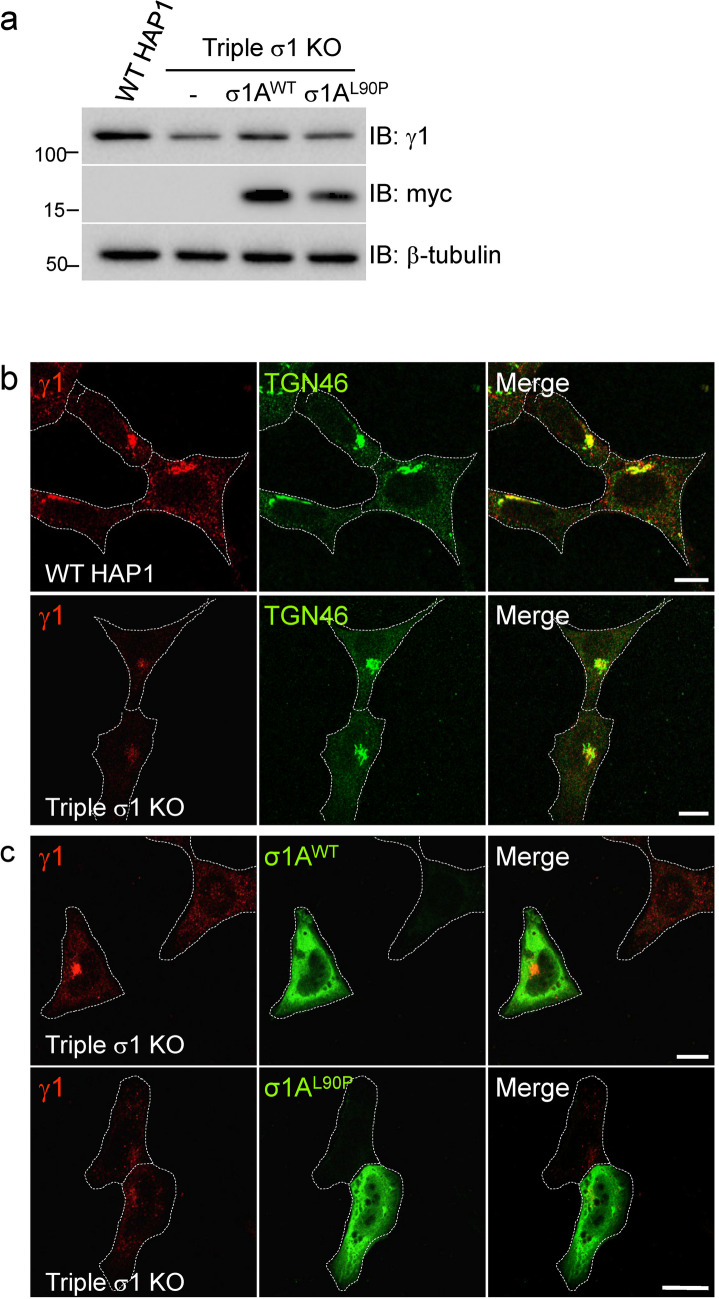
The σ1A L90P substitution prevents rescue of the association of AP-1 with TGN/endosomes in triple σ1-KO cells. **a** IB analysis of cell lysates from WT HAP1 cells and triple σ1-KO HAP1 cells untransfected (-) or transfected with plasmids encoding myc-tagged WT or L90P σ1A. Notice the partial rescue of γ1 levels in triple σ1-KO cells expressing σ1WT but not L90P σ1A. IB with anti-β-tubulin is shown as loading control. **b** Confocal immunofluorescence microscopy of WT and triple σ1-KO HAP1 cells stained for endogenous γ1 (red channel) and the TGN marker TGN46 (green channel). Notice the co-localization of γ1 and TGN46 in the perinuclear region of WT HAP1 cells, and the marked reduction in γ1 signal in the triple σ1-KO cells. **c** Confocal immunofluorescence microscopy of triple σ1-KO HAP1 cells transfected with plasmids encoding myc-tagged WT or L90P σ1A, and stained for endogenous γ1 (red channel) and the myc epitope (green channel). Notice the rescue of perinuclear γ1 immunostaining by expression of WT but not L90P σ1A. In b and c, cell edges are indicated with dashed lines; scale bars: 10 μm

Next, we investigated the effect of the σ1A L90P substitution on the intracellular localization of AP-1 by rescue of HAP1 cells having knock-out (KO) of all three σ1 isoforms (σ1A, σ1B and σ1C). We observed that KO of all three σ1 isoforms markedly reduced the levels of the γ1 and μ1A proteins, as analyzed by immunoblotting (Supplementary Fig. 2a, b). Expression of myc-tagged σ1A^WT^ in these cells resulted in partial restoration of endogenous γ1 levels, whereas expression of myc-tagged σ1A^L90P^ led to minimal or no recovery relative to untransfected cells (Fig. 3a). We also examined the effect of σ1A^L90P^ expression on the rescue of AP-1 by immunofluorescence microscopy. Triple KO of σ1 isoforms dramatically reduced TGN/endosomal staining for γ1 (Fig. 3b). Expression of myc-tagged σ1A^WT^ rescued TGN/endosomal γ1 staining, whereas expression of myc-tagged σ1A^L90P^ did not (Fig. 3c). These data indicated that the σ1A L90P substitution disrupts the association of γ1 with TGN/endosomes, likely due to impaired assembly of the whole AP-1 complex.

#### Impact of σ1A^L90P^ on sorting of tight-junction proteins

**Fig.4:**
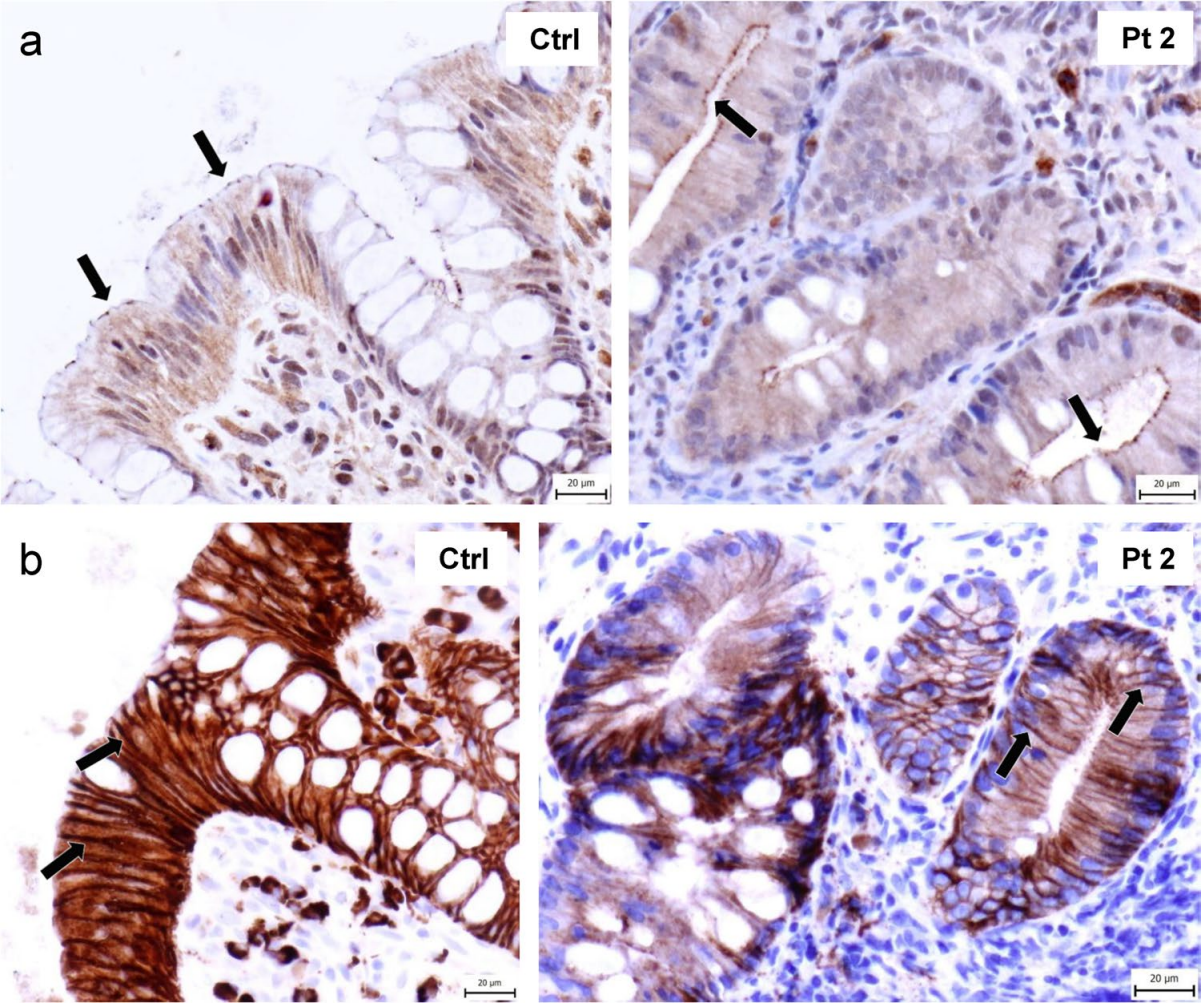
Immunohistochemical analysis of patient samples. **a** Immunohistochemical staining of ZO-1 in intestinal biopsy of healthy control and patient 2. Arrows show apical localization of the protein. **b** Immunohistochemical staining of claudin-3 in intestinal biopsy of healthy control and patient 2. Arrows show basolateral localization of the protein

Klee et al. showed that σ1A^L90P^-mediated disruption of AP-1 complex function leads to mislocalization of two tight junction proteins, ZO-1 and claudin 3, in a Caco-2-cellular model of the intestinal barrier [13]. However, due to the lack of material, they could not confirm the results on primary patient samples. We evaluated the localization of these two tight-junction proteins in intestinal biopsies of patient 2. Surprisingly, immunohistochemical staining of both ZO-1 and claudin 3 with the same antibodies used by Klee et al. showed correct localization of these proteins to the apical and basolateral plasma membrane, respectively, with no significant difference compared to the staining pattern of healthy controls (Fig. 4a, b).

Since it has been speculated that other σ1 isoforms could partially compensate for the dysfunctional σ1A isoform in vivo, we evaluated mRNA expression of *AP1S1*, *AP1S2* and *AP1S3* genes in FFPE samples of patient 2 taken from the duodenum and rectum at three different time points during the course of the disease. We detected expression of all three σ1 isoforms but no significant changes in the expression of the *AP1S2* and *AP1S3* isoforms in the patient samples compared to the healthy controls (Supplementary Fig. 3).

Taken together, the functional experiments demonstrated that the σ1A^L90P^ variant is virtually unable to assemble into the AP-1 complex, resulting in abrogation of DE]XXXL[LI]-signal recognition and drastic reduction in association of γ1 with the TGN/endosomes.

## Discussion

Similarly to other coatopathies, a substantial variability in the onset and severity of MEDNIK syndrome symptoms has been documented, especially in the case of enteropathy. This phenotype can vary even among carriers of the same AP-1 σ1A variant. While some patients die from dehydration in the neonatal period, others only exhibit symptoms later in life. Furthermore, the intestinal epithelium of MEDNIK syndrome patients is probably capable of a certain degree of maturation after microbial colonization of the intestine is completed, as observed in patient 2. Supportive care and genetic background may also play a role in this variability.

Although enteropathy, regardless of its severity, becomes apparent during the neonatal period, other symptoms of MEDNIK syndrome (e.g., neuropathy) develop later in life, as is the case for patient 2 in our study. Without previous knowledge of the diagnosis from other affected family members, establishing the diagnosis in the neonatal period can be complicated. This is especially true for patients with severe enteropathy who die shortly after birth and for which other symptoms may be overlooked or misattributed to malnutrition and infectious complications. MEDNIK syndrome patients might therefore be underreported among patients with severe neonatal onset enteropathy.

Significant effort has been made in the past to decipher the molecular pathogenesis of MEDNIK syndrome. Martinelli et al. proposed that disturbances in copper metabolism are caused by disrupted trafficking of the two most important copper transporters, ATP7A and ATP7B [10]. Using a zebrafish model, Monpetit et al. showed that knockdown of *AP1S1* altered skin formation and reduced pigmentation via abnormal localization of laminin, a protein important for cell–cell adhesion [8]. Furthermore, Klee et al. demonstrated the role of variant σ1A in disrupting intestinal barrier integrity by mislocalization of the tight-junction proteins ZO-1 and Claudin-3 [13]. While these studies focused mainly on elucidation of the pathogenesis at the cellular or tissue levels, we examined the impact of the L90P variant on the assembly and localization of the AP-1 complex. We found that the L90P substitution decreases the expression levels of σ1A to about 56% and the assembly of σ1A into the AP-1 complex to 0–13% compared to WT. Moreover, the L90P substitution renders σ1A completely incapable of interacting with [DE]XXXL[LI] sorting signals, likely due to its inability to interact with γ1, which also participates in [DE]XXXL[LI]-signal recognition [4, 6, 21]. These defects could stem from the disruption of a critical α-helix by the conformational rigidity of the proline residue. Based on these studies, we conclude that the L90P substitution leads to a complete σ1A LOF.

Our analyses of the effect of the σ1A L90P substitution on [DE]XXXL[LI] signal recognition used as probes the well-characterized sequences ERAPLI and ERQPLL from the the cytosolic tails of LIMP-II and tyrosinase, respectively [4, 6, 22]. However, the [DE]XXXL[LI]-containing cargos whose missorting underlies the pathogenesis of MEDNIK syndrome remain to be identified. This aspect should be the focus of future studies in polarized epithelial cells and neurons, given the neuroepithelial nature of MEDNIK syndrome.

It is also of note that disorders caused by variants in other AP-1 subunits (e.g., β1 and γ1), which also disrupt AP-1 function, manifest with symptoms overlapping with MEDNIK syndrome, supporting the concept that AP-1 deficiency is responsible for the full range of MEDNIK symptoms [23–29]. Indeed, patients with variants in the *AP1B1* gene encoding β1 (KIDAR syndrome) display symptoms almost identical to those of MEDNIK syndrome. The only difference between the symptoms of *AP1B1*-related KIDAR and *AP1S1*-related MEDNIK syndromes is the presence of ophthalmological problems, such as keratitis and corneal scarring, in KIDAR patients, which have not been reported in MEDNIK patients [24–29]. On the other hand, patients with variants in the *AP1G1* gene encoding γ1 (Usmani-Riazuddin syndrome) predominantly exhibit neurological symptoms [23]. The different presentations of these diseases likely result from the expression of subunit isoforms that could compensate for the loss of the variant subunit, as discussed further below.

Our study is the first to analyze the impact of the σ1A L90P variant at the molecular level, contributing to the understanding of the molecular pathogenesis of MEDNIK syndrome. The scarcity of studies confirming these findings in patient samples primarily stems from the rarity of the disease and the critical condition of the patients, which results in limited availability of patient material. Since the clinical status of patient 2 enabled us to perform an intestinal biopsy, we aimed to confirm in patient samples the published data from experimental models of enteropathy. Specifically, we evaluated the subcellular localization of two tight junction proteins, ZO-1 and claudin 3. These proteins were previously shown to be mislocalized in the Caco-2 enterocytic cell line with *AP1S1* KO, and their normal localization could be restored by transfection of *AP1S1*^WT^ but not *AP1S1*^c.269 T>C^ variant [13]. In contrast to these findings in an experimental model, we did not see any obvious difference in the localization of ZO-1 or claudin 3 in the patient’s enterocytes as compared to those from healthy controls. Since the σ1A L90P variant leads to virtually complete LOF of the σ1A subunit isoform, the observed discrepancy between the experimental model and patient samples could be due to a compensatory mechanism involving replacement of the missing σ1A with another σ1 isoform. However, we did not detect significant differences in the levels of mRNA of all three σ1 isoforms between healthy and patient intestinal biopsies. Although these findings do not support the notion that σ1A deficiency may be compensated by other σ1 isoforms, it is important to consider technical limitations of mRNA analysis, as well as the current unavailability of isoform-specific antibodies to confirm this hypothesis at the protein level.

If indeed the missing σ1A function can be replaced by other σ subunits, one might speculate why Klee et al. did not detect such compensation in the Caco-2 cell line following the *AP1S1* KO. A possible explanation is that the levels of other σ1 isoforms in Caco-2 cells are insufficient to assemble the AP-1 complex. This hypothesis is supported by previously published RNA-Seq data showing lower expression of *AP1S2* and *AP1S3* compared to *AP1S1* mRNAs in Caco-2 cells [30]. In contrast, in the HAP1 cell line used in some of our experiments, the expression of *AP1S1* and *AP1S2* mRNA is comparable [31]. Accordingly, HAP1 cells with triple σ1 KO showed the highest decrease in the levels of γ1 and μ1 subunits of AP-1, whereas HAP1 cells with KO of only two σ1 isoforms exhibited less pronounced reductions, suggesting a partial redundancy of σ1 isoforms (Supplementary Fig.2 a, b).

While no causal treatment is currently available for MEDNIK syndrome patients, some of them have been reported to improve their clinical condition upon treatment with zinc acetate, a drug targeting copper overload [10]. This treatment was initiated in both of our patients once the diagnosis was established; however, no significant improvement in their symptoms was observed.

In summary, we present two additional unrelated patients with the *AP1S1* c.269 T > C variant who developed MEDNIK syndrome, and revise substantially the clinical characterization of patients with *AP1S1* missense variants. We also provide essential experimental evidence on the molecular pathogenesis of the σ1A L90P variant, showing its dramatic impact on the assembly and function of the AP-1 complex. Based on these results, we suggest classifying cases with *AP1S1* c.269 T > C as MEDNIK, and considering MEDNIK syndrome in the diagnostic workup of patients with severe early-onset enteropathy.

## Supplementary Information

Below is the link to the electronic supplementary material.

Supplementary file1 (DOCX 542 KB)

## Data Availability

The data that support the findings of this study are available from the corresponding authors upon reasonable request.
