# Supplementary material for: Revising pathogenesis of AP1S1-related MEDNIK syndrome: a missense variant in the AP1S1 gene as a causal genetic lesion
Source: J Mol Med (Berl). 2024 Sep 13;102(11):1343–53. doi: 10.1007/s00109-024-02482-0 (PMC11525306; doi:10.1007/s00109-024-02482-0)
Supplement: Supplementary file 1 — Supplementary file1 (DOCX 542 KB) [file 109_2024_2482_MOESM1_ESM.docx]

**SUPPLEMENTARY MATERIALS FOR**

**Revising Pathogenesis of *AP1S1*-Related MEDNIK syndrome: A Missense Variant in the *AP1S1* Gene as a Causal Genetic Lesion**

Marketa Rackova1, Rafael Mattera2, Michael Svaton1, Filip Fencl3, Veronika Kanderova1, Karolina Spicakova3, Sang Yoon Park2, Ondrej Fabian4, Miroslav Koblizek4, Eva Fronkova1, Juan S. Bonifacino2,#, Karolina Skvarova Kramarzova1,#

1CLIP, Department of Pediatric Hematology and Oncology, Second Faculty of Medicine, Charles University and University Hospital Motol, Prague, Czech Republic

2Section on Intracellular Protein Trafficking, Neurosciences and Cellular and Structural Biology Division, *Eunice Kennedy Shriver* National Institute of Child Health and Human Development, National Institutes of Health, Bethesda, MD, USA

3Department of Pediatrics, Second Faculty of Medicine, Charles University and University Hospital Motol, Prague, Czech Republic

4Department of Pathology and Molecular Medicine, Second Faculty of Medicine, Charles University and University Hospital Motol, Prague, Czech Republic

#These authors contributed equally

**Correspondence:** karolina.skvarova@lfmotol.cuni.cz and juan.bonifacino@nih.gov

**SUPPLEMENTARY METHODS**

**Antibodies**

The following antibodies were used: mouse anti-AP-1 γ1 (BD Biosciences, Franklin Lakes, NJ, USA, cat. 610385), rabbit anti-AP-1 μ1A (Proteintech, Rosemont, IL, USA, cat. 12112-1-AP), rabbit anti-AP-1 σ1A (Bethyl Laboratories, Montgomery, TX, USA, cat. A305-396A), rabbit anti-myc tag (Cell Signaling Technology, Danvers, MA, USA, cat. 2272), sheep anti-TGN46 (Bio-Rad, Hercules, CA, USA, cat. AHP500G), rabbit anti-β-tubulin (Cell Signaling Technology, cat. 2146), Alexa Fluor 488- conjugated donkey anti-sheep IgG (Thermo Fisher Scientific, Waltham, MA, USA, cat. A-11015), Alexa Fluor 555- conjugated donkey anti-mouse IgG (Thermo Fisher Scientific, cat. A-31570), HRP-conjugated goat anti-rabbit IgG (Santa Cruz Biotechnology, Dallas, TX, USA, cat. sc-2030), and HRP-conjugated goat anti- mouse IgG (Santa Cruz Biotechnology, cat. sc-2031).

**Immunoprecipitation and immunoblotting**

HEK293T cells transiently transfected with plasmids encoding myc-tagged σ1AWT or σ1AL90P were washed with PBS and lysed in ice-cold lysis buffer (0.5% Triton X-100, 50 mM Tris-HCl pH 7.5, 150 mM NaCl, 5 mM EDTA), supplemented with a protease inhibitor cocktail (Sigma-Aldrich, Burlington, MA, USA). Cell lysates were spun for 15 min at 20,000 x *g*, and supernatants incubated with antibodies to the myc epitope or the AP-1 γ1 subunit bound to protein G-Sepharose beads (Amersham Biosciences, Slough, United Kingdom) for 1 h at 4°C. Beads were washed 3 times with 10 mM Tris-Cl pH 7.5, 150 mM NaCl, 0.5 mM EDTA, and eluted with 1X Laemmli sample buffer at 95°C for 5 min. Samples were subjected to SDS-PAGE, transferred to a nitrocellulose membrane, and sequentially probed with the indicated primary antibodies and corresponding secondary antibodies conjugated to HRP. Immunoblots were developed with SuperSignal West Dura (Thermo Fisher Scientific) and imaged with a ChemiDoc MP imaging system (Bio-Rad). For immunoblotting of HAP1 cells, the cells were washed with PBS and lysed with 1% Triton X-100, 300 mM NaCl in 50 mM Tris-HCl pH 7.4 for 30 min on ice. Lysates were centrifuged for 10 min at 17,000 x *g* at 4 ̊C, and supernatants collected. Total protein concentration was measured and normalized using Bradford reagent (Sigma-Aldrich). Samples were supplemented with 1X Laemmli sample buffer and heated at 95°C for 5 min, and analyzed by SDS-PAGE and immunoblotting as described above. Band intensity after background subtraction was quantified by image analysis using Fiji (https://fiji.sc/).

**Immunofluorescence microscopy**

HAP1 cells were fixed with 4% paraformaldehyde in 0.1 mM CaCl2, 1 mM MgCl2, and PBS (PBSCM) for 15 min at room temperature. Cells were permeabilized with 0.2% Triton X-100 in PBSCM for 10 min at room temperature and sequentially incubated with primary and secondary antibodies in PBSCM containing 0.2% BSA either overnight at 4 ̊C or for 1 h at 37 ̊C. Cells were washed in PBSCM and mounted with DAPI-Fluoromount-G (Electron Microscopy Sciences, Hatfield, PA, USA). Microscopy images were acquired using a Zeiss 780 confocal microscope with a Plan Apochromat 63x objective (N.A. 1.40).

**Immunohistochemistry**

Tissue samples were formalin-fixed and paraffin-embedded during the routine diagnostic procedure. The most representative specimens of colic mucosa from each endoscopic examination were selected for immunohistochemical studies. Immunohistochemical staining was performed with antibodies to the following proteins: ZO-1 (Thermo Fisher Scientific, cat. 61- 7300, dilution 1:3,000) and claudin 3 (Sigma-Aldrich, cat. SAB4500435, dilution 1:3,000). An intestinal biopsy sample from the unaffected part of the intestine of a young patient with intermittent diarrhea but no endoscopic or histopathological findings was used as healthy control tissue.

**Recombinant DNA constructs**

A pcDNA 3.1 (hygro +)-based vector harboring a cDNA encoding human σ1A with a C-terminal triple-myc tag inserted at the BamH1/XbaI sites [1] was used to transiently transfect HeLa, HEK293T and HAP1 cells. Yeast three-hybrid (Y3H) constructs pBridge-human LIMP-II tail.human AP-1 σ1A and pBridge-mouse tyrosinase tail.human AP-1 σ1A, with LIMP-I or tyrosinase tails subcloned in the multiple cloning site (MCS) 1 (MCS1) and AP-1 σ1A in the MCS2 of the GAL4 DNA binding domain (BD) vector pBridge (Clontech, Mountain View, CA, USA), were previously described [2, 3]. Y3H constructs encoding mouse AP-1 γ1, rat AP-2 αC or human AP-3 δ subunits subcloned in the GAL4 transcriptional activation domain (AD) vector pGADT7 (Clontech) were also previously described [3]. Substitutions to generate the corresponding AP-1 σ1A L90P mutants were generated by site-directed mutagenesis (QuikChange, Agilent, Santa Clara, CA, USA) of the WT constructs. All constructs were verified by Sanger sequencing.

**Yeast three-hybrid (Y3H) assays**

Y3H assays were performed using the HF7c reporter strain as described [3]. Yeast double transformants were plated in medium lacking leucine, tryptophan and methionine but containing histidine (+His) to control for viability and loading, and in the same medium lacking histidine (-His) to detect protein interactions. Positive and negative controls used in the assays are described in the corresponding figure legend.

**Gene expression assays**

Total RNA from FFPE samples was reverse-transcribed to cDNA using High-Capacity RNA-to- cDNA^TM^ Kit (Applied Biosystems, Waltham, MA, USA). Gene expression of the σ1 isoforms and *GAPDH* was determined using TaqMan Gene Expression Assays (*AP1S1* - Hs00933242_m1; *AP1S2* - Hs00993537_g1; *AP1S3* - Hs00950999_m1; *GAPDH* - Hs99999905_m1, all from Thermo Fisher Scientific) on a 7500 Fast Real-Time PCR System instrument (Applied Biosystems). The mRNA expression of σ1 isoforms in the samples of patient 2 (three consecutively obtained samples from duodenum and rectum) and six healthy controls (three samples of unaffected duodenum and three samples of unaffected rectum) was normalized to *GAPDH* expression using the delta Ct method. Results were analyzed using the statistical software Prism (GraphPad, La Jolla, CA, USA). Differences between the patient and control samples were evaluated by non-parametric Mann-Whitney test; p-value less than 0.05 was considered significant.

**Knock-out of AP-1 σ1**-**subunit genes by CRISPR/Cas9**

To generate double σ1A/B and σ1A/C KO HAP1 cells, two gRNA sequences (5’- ATGCTATTATTCAGCCGGCA-3’, 5’-GATGGTGCGCGAGCTCATGC-3’) targeting the first exon of the *AP1S1* gene were subcloned into pSpCas9(BB)-2A-GFP (pX458, a gift from Feng Zhang (Addgene plasmid # 48138)) and transfected into either σ1B KO or σ1C KO HAP1 cells (Supplementary Fig. 4a-c). Cells transfected by pX458-gRNA constructs were sorted for GFP by fluorescence-activated cell sorting (FACS). Genomic DNA sequencing identified clones predicted to bear a deletion of amino acids 10-35 of σ1A in the σ1B KO background, and a frameshift causing premature termination of σ1A after amino acid 33 in the σ1C KO background (Supplementary Fig. 4c). For the triple σ1Α-σ1Β-σ1C KO, two gRNA sequences (5’- ATATTGCTCTTCAGTCGACA-3’, 5’-GTTCAGATTATTCTCTCCCG-3’) targeting the second exon of the *AP1S3* gene were subcloned into the pX458 construct, and introduced into the σ1A-σ1B double KO background. Cells transfected with pX458-gRNA constructs were sorted for GFP by FACS. Genomic DNA sequencing identified a clone with a 98-nt deletion causing frame shift and premature termination after amino acid 17 in one allele, and a 99-nt deletion causing removal of amino acids 11-43 in the other allele (Supplementary Fig. 4d).


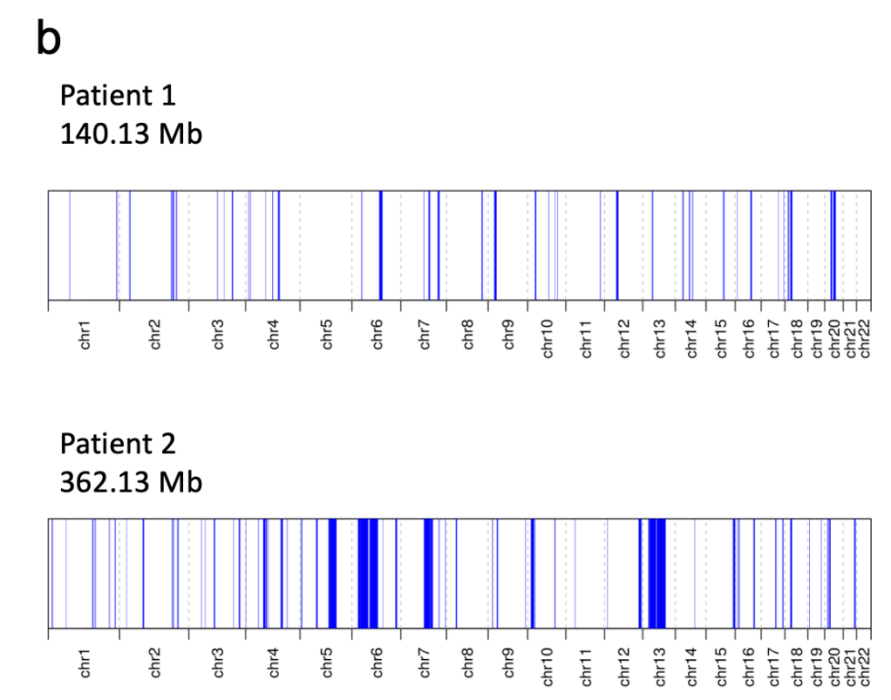

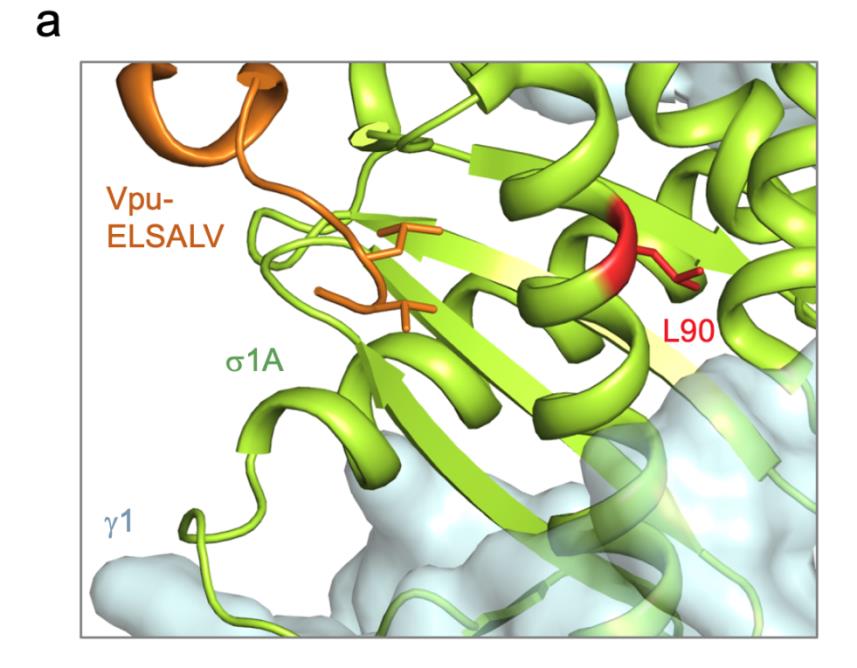
**SUPPLEMENTARY FIGURES**

**Supplementary Fig.1 Position of the L90 residue in the AP-1 σ1A crystal structure and heterozygosity mapping analysis of patients**

(a) Detail of the crystal structure of AP-1 with a dileucine motif (ELSALV) adapted from Jia et al. 2014 [4] (PDB ID: 4P6Z) and rendered using PyMol (https://pymol.org/). The AP-1 σ1A L90 residue is on an α-helix that is part of the dileucine-binding pocket, although its side chain faces opposite to the pocket. (b) Runs of Homozygosity (ROH) analysis by AutoMap Tool showing high content of homozygous regions in both patients.


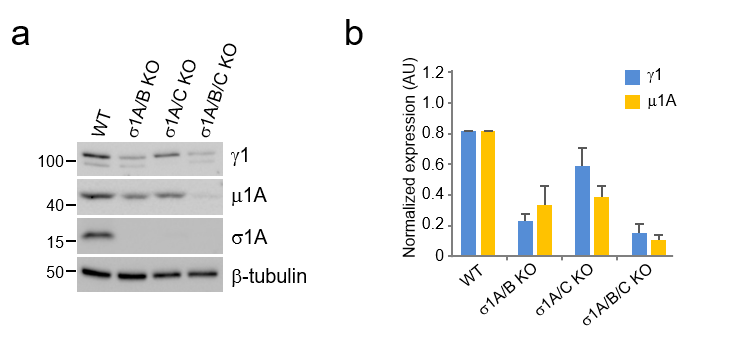


**Supplementary Fig.2 Reduction of levels of AP-1 complex subunits upon KO of σ1 isoforms**

(a) Immunoblot analysis for AP-1 subunits in the double or triple σ1 KO HAP1 cells. The positions of molecular mass markers (in kDa) are indicated on the left. (b) Quantification of immunoblot analysis data for γ1 and μ1A from experiments such as that shown in a (mean ± SEM; n=3)


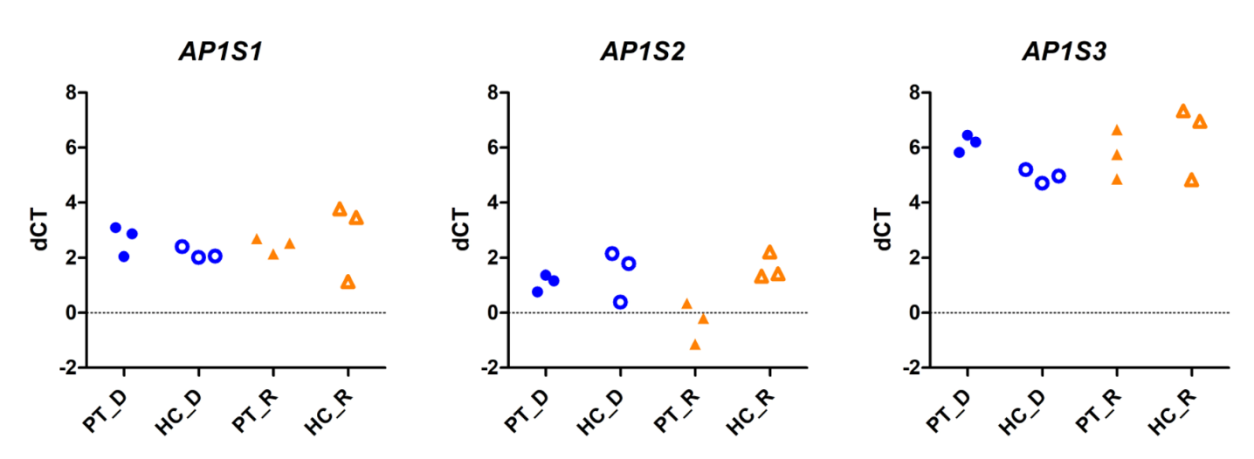


**Supplementary Fig.3 qPCR of different σ1 subunit isoforms**

Consecutively obtained samples from duodenum (N=3) and rectum (N=3) of patient 2 and samples of healthy controls (duodenum (N=3) and rectum (N=3)) were analyzed. The results are depicted as dCT of each isoform. No significant differences in the expression of σ1 isoforms between healthy and patient intestinal tissue were detected. PT = patient 2, HC = healthy control, D = duodenum, R = rectum


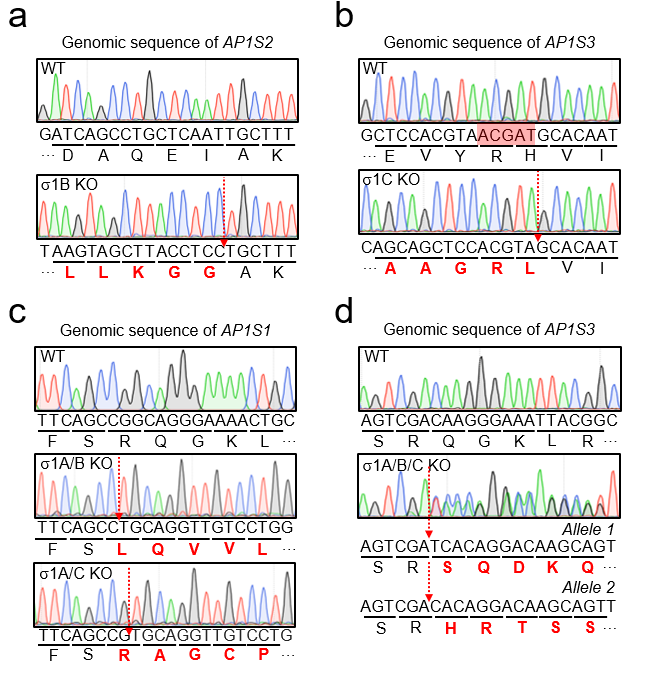


**Supplementary Fig.4 Sanger sequencing of genomic DNA from WT and σ1 KO HAP1 cells**

(a) Genomic DNA sequence of σ1B-KO HAP1 cells. The red arrow shows the position of a 23-nt deletion in exon 4 of the *AP1S2* gene causing premature termination after amino acid 136. (b) Genomic DNA sequence of σ1C-KO HAP1 cells shows a 5-nt deletion in exon 3 of the *AP1S3* gene (shadowed in red in WT sequence) causing open reading frame shift and premature termination after amino acid 97. (c) Genomic DNA sequence of σ1A-σ1B double KO HAP1 cells (middle sequence) shows a 78-nt deletion in exon 2 of the *AP1S1* gene causing a deletion of amino acids 10-35. Genomic DNA sequence of σ1A-σ1C double KO HAP1 cells (bottom sequence) shows a 77-nt deletion in exon 2 of the *AP1S1* gene causing open reading frame shift and premature termination after amino acid 33. (d) Genomic DNA sequence of σ1A-σ1B-σ1C triple KO HAP1 cells (three bottom sequences) shows a 98-nt deletion in exon 1 of the *AP1S3* gene causing open reading frame shift and premature termination after amino acid 17 (Allele 1) or a 99-nt deletion resulting in removal of amino acid 11-43 (Allele 2). The sequences of WT *AP1S1, AP1S2* and *AP1S3* are shown at the top of panels for comparison purposes

**SUPPLEMENTARY REFERENCES**

1. Jain S, Farías GG, Bonifacino JS (2015) Polarized sorting of the copper transporter ATP7B in neurons mediated by recognition of a dileucine signal by AP-1. Mol Biol Cell 26:218–228. https://doi.org/10.1091/MBC.E14-07-1177

2. Janvier K, Kato Y, Boehm M et al (2003) Recognition of dileucine-based sorting signals from HIV-1 Nef and LIMP-II by the AP-1 gamma-sigma1 and AP-3 delta-sigma3 hemicomplexes. J Cell Biol 163:1281–1290. https://doi.org/10.1083/JCB.200307157

3. Mattera R, Boehm M, Chaudhuri R et al (2011) Conservation and diversification of dileucine signal recognition by adaptor protein (AP) complex variants. J Biol Chem 286:2022–2030. https://doi.org/10.1074/JBC.M110.197178

4. Jia X, Weber E, Tokarev A et al (2014) Structural basis of HIV-1 Vpu-mediated BST2 antagonism via hijacking of the clathrin adaptor protein complex 1. Elife 3:e02362. https://doi.org/10.7554/ELIFE.02362
